# Fostering Formal Commutativity Knowledge with Approximate Arithmetic

**DOI:** 10.1371/journal.pone.0142551

**Published:** 2015-11-11

**Authors:** Sonja Maria Hansen, Hilde Haider, Alexandra Eichler, Claudia Godau, Peter A. Frensch, Robert Gaschler

**Affiliations:** 1 University of Cologne, Department of Psychology, Cologne, Germany; 2 Humboldt-Universität zu Berlin, Exzellenzcluster Bild Wissen Gestaltung, Berlin, Germany; 3 FernUniversität in Hagen, Department of Psychology, Hagen, Germany; Center for BrainHealth, University of Texas at Dallas, UNITED STATES

## Abstract

How can we enhance the understanding of abstract mathematical principles in elementary school? Different studies found out that nonsymbolic estimation could foster subsequent exact number processing and simple arithmetic. Taking the commutativity principle as a test case, we investigated if the approximate calculation of symbolic commutative quantities can also alter the access to procedural and conceptual knowledge of a more abstract arithmetic principle. Experiment 1 tested first graders who had not been instructed about commutativity in school yet. Approximate calculation with symbolic quantities positively influenced the use of commutativity-based shortcuts in formal arithmetic. We replicated this finding with older first graders (Experiment 2) and third graders (Experiment 3). Despite the positive effect of approximation on the spontaneous application of commutativity-based shortcuts in arithmetic problems, we found no comparable impact on the application of conceptual knowledge of the commutativity principle. Overall, our results show that the usage of a specific arithmetic principle can benefit from approximation. However, the findings also suggest that the correct use of certain procedures does not always imply conceptual understanding. Rather, the conceptual understanding of commutativity seems to lag behind procedural proficiency during elementary school.

## Introduction

Mathematical principles guide our daily lives. Understanding quantitative relations is one of the most important abilities that enable us to act independently in modern societies. Without it, we would not be able to estimate our expenses when we go shopping, nor could we coordinate our time with our daily obligations. Therefore, it is important for children to cultivate mathematical competencies by acquiring mathematical concepts and procedures. But there is still the need to investigate how children acquire early procedural and conceptual knowledge of mathematical principles and how this knowledge might develop over time.

In line with Hiebert and LeFevre [1], we define procedural knowledge (or ‘knowing how’) as the ability to apply a particular strategy in a specific problem context. Conceptual knowledge (or ‘knowing why’ [1]) refers to an abstract understanding of the principle underlying the boundary conditions for applying a procedure. According to this definition, correctly applying a certain procedure while solving a mathematical problem reflects procedural knowledge (knowing how). It does not necessarily imply that the problem solver also possesses conceptual knowledge, in the sense that he/she also knows why and under which conditions the procedure works [2–6]. As Baroody ([7], p.27) puts it, “computational efficiency can be achieved without understanding”.

Based on the assumption that children acquire precursory mathematical knowledge before entering school [8–9], the main goal of the current study was to investigate if a reference to such early levels of mathematical understanding can alter the access to procedural and/or conceptual knowledge about specific mathematical principles.

### 1.1 From concrete to abstract instantiations

The development of procedural and conceptual knowledge, as well as their interrelations, is one of the leading questions in the field of mathematical development [10–12]. One related question concerns the different levels on which children understand these aspects [9, 13]. In her model of mathematical thinking, Resnick [9] proposed that children already acquire some mathematical abilities long before entering school. These early abilities are object-bound and concrete before they then become more and more abstract representations. On the object-bound level, children can successfully perform operations like comparison or combination of physical objects but not of abstract numbers. They can only do this approximately, rather than on the level of exact quantification. For instance, children on this level might know that the tennis ball is smaller than the football. They might also possess some precursory knowledge about arithmetic principles. Arithmetic principles are fundamental laws or regularities within a given problem domain [14]. These principles can range from general and simple, like “addition makes more”, to increasingly abstract principles like commutativity [15–16]. At the object-bound stage, the understanding of the first principle could already be observed in 5-month-old infants. Wynn [17], for instance, found they look at 'impossible outcomes' of addition problems longer than at possible outcomes. An example of an impossible outcome would be the addition of one doll to another behind an occluder resulting in only one doll. But there are also precursors of the more abstract principles. In the case of commutativity, children might know that it does not matter whether you get the red or the blue candy first, as long as you get both samples. While not being able to represent the exact quantities, they might thus know about irrelevance of order of added quantities.

When we speak of 'principles' we refer specifically to abstract principles that enable learners to use time-saving strategies and shortcuts. In line with Resnick’s [9] assumptions, several studies suggest that children already show signs of procedural as well as conceptual knowledge, even of abstract mathematical principles like, for instance, commutativity long *before* they enter school and receive first formal instructions [9, 13, 18–23]. In particular, toddlers perform well above chance as long as they are confronted with concrete material or if they are only supposed to estimate rather than exactly compute the solutions for symbolic problems [9, 24–26]. Thus, children acquire some mathematical knowledge without any formal instruction [27]. This early-acquired knowledge is probably best understood as a pool of precursory mathematical concepts. Dehaene [8] assumes that this kind of knowledge might be based on an evolutionary old quantity representation system, the so-called approximate number system (ANS [28–32]). Knowledge representations in this system apparently develop independent of culture and language. However, it is not entirely clear how children might link their precursory knowledge to exact arithmetic knowledge as it is required by formal instructions in school.

According to current practice in school, precursory knowledge is usually not actively used to foster the acquisition of formal knowledge. The longer they have attended school, children instead tend to increasingly separate their precursory mathematical knowledge acquired in real world contexts from formal mathematical understanding (see e.g. [33–35]). One way to avoid this phenomenon might be to explicitly rely on such precursory mathematical knowledge when introducing new arithmetic concepts in school (e.g. [36–37]). For instance, several studies provide evidence that relying on children’s ability of approximate calculation also facilitates their exact calculation competencies [24–25, 28–29]. Recently, Hyde, Khanum, and Spelke [38] trained children with nonsymbolic approximate addition and number comparison problems. Subsequently, they let children work through an exact symbolic addition task. Compared to two different control conditions with other training tasks, the short approximate calculation training significantly improved the children´s performance in the subsequent exact symbolic addition task.

These findings raise the question of whether the activation of such precursory mathematical knowledge can also enhance the understanding of abstract mathematical principles like, for instance, equivalence problems or the commutativity principle. First results come from Sherman and Bisanz ([39], see also [37]). They investigated the effect of concrete, nonsymbolic material on the understanding of equivalence problems in formal arithmetic. They first instructed second graders to solve nonsymbolic equivalence problems and afterwards symbolic equivalence problems. In a second condition, students received the reverse order. The results revealed that solving nonsymbolic problems first facilitated the performance in symbolic problems, whereas symbolic problems did not affect the performance in nonsymbolic problems.

Thus, recent findings suggest that activating children’s precursory knowledge by presenting nonsymbolic problems or approximate calculation problems can positively influence their performance of exact symbolic arithmetic. However, up to now only few studies have investigated whether approximate calculation also enhances the understanding of less basic arithmetic principles. On the one hand, approximate calculation might activate existing precursory conceptual knowledge that is useful in later exact calculation. On the other hand, approximate calculation might help on a more general path by promoting flexibility in problem solving, thus requiring and triggering procedural knowledge. The latter seems more likely because acceptable estimation results can be reached by diverse procedures [16, 40]. Presumably, triggering flexibility by applying an approximation task can spill over to calculation tasks presented afterwards (see also [38]). Here, we test if activating mathematical knowledge of an arithmetic principle in approximate calculation problems boosts using the same knowledge in exact arithmetic problems. As a rather direct take on this link, we used a symbolic format in approximation similar to that used by Gilmore et al. [24].

### 1.2 The current study

The main goal of the current experiments was to examine if approximately calculating the results of symbolic problems that link to a specific mathematical principle can alter children’s ability to spontaneously spot and use this arithmetic principle in exact arithmetic problems. A second goal was to test whether alluding to a principle in approximation affects only procedural or additionally also the conceptual understanding of the principle.

We used the commutativity principle as a test case. Its core property, the order-irrelevance principle, is ubiquitous in everyday situations. The commutativity principle states that in binary operations of addition or multiplication, the order of the operands does not affect their sum or product (cf. a + b = b + a; see [15]). Children can experience the core principle of order-irrelevance in many non-numerical, as well as numerical, everyday situations long before entering school. For instance, a child may learn that the order is irrelevant when laying the table or when putting on one’s socks. By contrast, putting on underpants and trousers clearly does require a strict order. Consequently, already toddlers might know that order is irrelevant in some situations and relevant in others. If order is irrelevant, they also learn that combining two different sets of objects leads to the same result regardless of the order (e.g. [9, 19–20, 22–23]). Moreover, when children start to compute simple addition problems at the age of 4 to 5 years, they often spontaneously use the min-strategy. That is, they start counting up from the larger addend even if the smaller addend was presented first [13, 18–21]. Hence, there is good evidence that even preschoolers already possess precursory knowledge about commutativity. This is in line with data reported by Dowker [41–42] who compared the derived fact strategy use in 6–7 year-olds. Derived fact strategies refer to the ability to extract new arithmetic facts from known facts on the basis of arithmetic principles like commutativity, associativity or the inversion principle [16]. In Dowker’s study, children had to solve addition and subtraction problems slightly too difficult for them to compute, on the basis of a previously given result of a related problem. The relationship between the two problems consisted of a specific arithmetic principle. Among several principles, Dowker found commutativity to be the one used the second-most. Only the basic identity principle (understanding that the exact repetition of an arithmetic problem will result in the same total) was even easier for the participants.

Most studies on commutativity assess children’s knowledge about this principle by asking them to solve an arithmetic problem first and then to describe their strategy [18, 23, 43]. For example, Canobi, Reeve, and Pattinson [44] told children to solve addition problems, interspersed with commutative ones. After a child had solved a problem, the interviewer asked how she/he “worked out the answer”, and prompted her/him when necessary. For instance, children who counted were asked, “What was the first number you said as you started counting?” They assumed that the children had used their conceptual knowledge of commutativity if they reported solving a problem by referring to a related, immediately preceding problem, for instance, "I saw that 2 + 7 had the same numbers as 7 + 2 (the preceding problem), so I knew the answer to 2 + 7 was 9 as well" [44]. This combined assessment of procedural and conceptual knowledge enables researchers to investigate if a child only applies the strategy (procedural knowledge) or if he/she additionally understands why the strategy applies [1, 3, 7, 23, 45]. However, it is unclear whether asking children to explain their solution strategies might trigger the use of shortcut strategies during the investigation. It is conceivable that children look at the problems more attentively and select strategies more flexibly when they are asked to verbalize their procedures. Consequently, conclusions concerning the question of whether a child is able to spontaneously use her/his knowledge about a certain mathematical principle might vary depending on the tests that were applied. On that note, Schneider and Stern [46] called for assessing procedural and conceptual knowledge in the context of arithmetic development multifaceted and independently of each other (see also [6]).

Here, we wanted to test if experiencing the commutativity principle in approximate calculation first will foster the *spontaneous* exploitation of the commutativity principle in exact arithmetic problems. Therefore, we took a slightly different approach to assess procedural and conceptual commutativity knowledge in exact arithmetic calculation [47]: first, we never informed the children about the existence of commutative problems. Second, we used two different task types in order to assess their procedural and conceptual knowledge separately. Both tests were presented in a school-like situation. The so-called *computation task* was aimed at assessing procedural knowledge. The *judgment task* served to measure children’s conceptual knowledge.

In the computation task, children received two subsets of problems; one subset contained commutative problems, the other did not. Time to work through each subset was limited so that it was very unlikely for the children to solve all problems. Children were explicitly told that it was impossible to solve all problems of a subset within the given time to prevent a loss of motivation. Importantly, this time limit enabled us to compare the number of solved problems between the two subsets. If children rely on the timesaving commutativity-based shortcut (that is, writing down the solution of a commuted problem without calculating anew), they should solve more problems per time in the subset containing commutative problems compared to the one that lacks such shortcut options.

The logic for assessing conceptual knowledge was similar: in the *judgment task*, children received commutative and noncommutative problems without any further information. They were simply asked to mark those problems which they believed required no calculation in order to get to the result. If children possess conceptual knowledge, they should be able to figure out that this only applies to commutative problems (see [48]). We assume that the judgment task taps children’s metacognitive knowledge of the commutativity principle which according to Flavell [49] is an essential component of conceptual understanding. Thus, this task allowed us to assess conceptual knowledge about commutativity without informing children about the existence of commutative problems.

**Fig 1:**
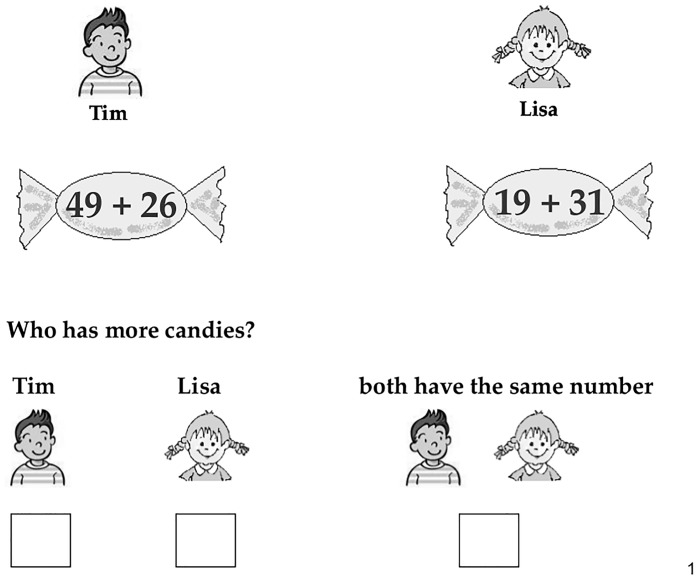
Example of a noncommutative trial presented in the approximation task.

**Fig 2:**
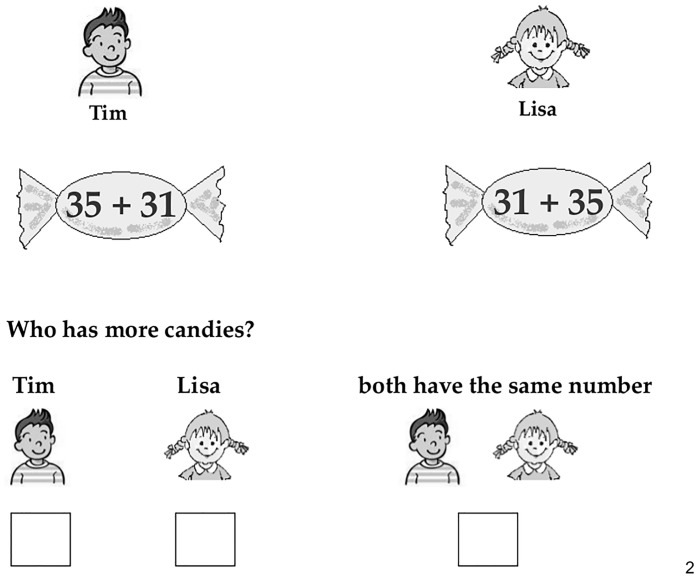
Example of a commutative trial presented in the approximation task.

Before children received these two exact arithmetic tasks, half of them were administered the induction task: the approximate arithmetic task. This task was similar to the symbolic approximate arithmetic task used by Gilmore et al. [24]. Each problem presented the pictures of two children (Tim and Lisa) who possessed a large candy, respectively. Each candy contained a symbolic addition problem composed of two two-digit addends (e.g. Tim’s candy contained “35 + 31” and Lisa’s candy “31 + 35” see Figs 1 and 2). Children were asked to judge whether both, Tim and Lisa, possessed the same amount of candy or whether either Tim or Lisa had more candy. The induction task contained commutative and noncommutative problems as well.

In Experiment 1, we tested first-graders who had not received any formal classroom instruction about the commutativity principle yet. Thus, from the perspective of formal mathematics instruction, these children were entirely unfamiliar with the principle of commutativity. In Experiment 2 and 3, we then investigated if the positive influence of the approximate calculation task on commutativity knowledge found in Experiment 1 is restricted to procedural knowledge or extends to the activation of conceptual understanding of commutativity.

## Experiment 1

The main goal of Experiment 1 was to investigate whether children who had not yet received any formal instruction about the commutativity principle would benefit from approximate calculation problems with respect to spontaneously spotting and applying commutativity-based shortcut options in exact arithmetic problems. For this purpose, we investigated first graders who had attended school for approximately four months and had not yet learned about commutativity in school. Half of the children started with the approximate symbolic arithmetic problems (*approximation task*, hereafter) and then received the exact arithmetic problems (*approximation-first group*). The remaining children were administered to the reversed order of tasks; that is, they solved the exact arithmetic problems first (*computation task*, hereafter) and then the approximation task (*computation-first group*). If the approximation task triggers the exploitation of commutativity in the exact arithmetic problems, children in the approximation-first group should show a larger commutativity benefit than the computation-first group.

### 2.1 Method

#### Ethic Statement

Only children whose parents or guardians had given the teacher their written consent participated in our study. For the participation in studies based on typical teaching methods and curricula, no special permission is required in Germany, so IRB approval was not necessary. All procedures were performed in full accordance with German legal regulations and the ethical guidelines of the DGPs (Deutsche Gesellschaft für Psychologie—German Society for Psychology) [50].

#### Participants

Sixty-eight (43 girls) first graders with a mean age of 6 years and 8 months (*SD* = 5.3 months) who had been attending school for four months took part in our study. We recruited them from one elementary school in a middle socio-economic status suburb of Cologne. All children had permission to take part in our study and were assigned evenly to the two experimental conditions. Thirty-six children (24 girls) participated in the approximation-first group, 32 children (19 girls) in the computation-first group.

### 2.2 Materials and Procedure

#### Materials

The study consisted of two different tasks, the approximation task and the computation task. Both tasks were conducted in one session lasting approximately 45 min. The *approximation task* was primarily used to trigger the exploitation of commutativity in exact arithmetic problems. Therefore, it contained only 11 pairs of two-digit addition problems that were either commutative (i.e., the order of the addends in the first problem was reversed in the second problem) or noncommutative. In each of these 11 trials, the pictures of two children (Tim and Lisa) were shown together with a large candy. The respective candy symbolized the number of candies of Tim and Lisa. To this end, each candy contained a symbolic addition problem composed of two addends larger than 10 (e.g. Tim’s candy contained “35 + 31” and Lisa’s candy “31 + 35” see Figs 1 and 2). The addends ranged between 13 and 97 leading to results between 36 and 140. Children were asked to estimate if both Tim and Lisa possessed the same number of candies or whether Tim or Lisa had more candies. They were explicitly told to estimate, and not to engage in exact calculation.

Seven out of the 11 trials contained commutative problem pairs: the candies of Lisa and Tim contained identical addends in different order (“23 + 45” and “45 + 23”). The remaining four trials were noncommutative. We constructed the results in both candies in a ratio that first graders can discriminate [24–25, 30]. For each noncommutative trial, we used one of the three ratios 6:10, 6:9 and 6:8 for the two results to be compared. For instance, the two problems “38 + 28” and “42 + 57” lead to the totals of 66 and 99, resulting in a ratio of 6:9. These large ratios should minimize the risk that participants mistakenly judge the two addition problems as commutative when they are indeed noncommutative. In half of the noncommutative problems, Tim possessed the larger amount of candies whereas Lisa did in the other half. In addition, in half of all the problems (commutative and noncommutative), the larger addend was the first one. This should discourage using heuristic shortcut strategies (e.g., comparing only the first addend of the two problems). Time limits and the large addends ensured that children did not calculate the results of these problems.

The *computation task* was composed of two subsets, the commutative and the noncommutative subset that contained 30 problems each. Both subsets were presented as small booklets of five pages with six problems on each page (see Table 1 for an example). In the commutative subset, two out of the six problems per page were commutative to the immediately preceding problem. This was the only difference between the commutative and the noncommutative subset. In both subsets, the problems consisted of two different addends between 1 and 9 (maximum result was 17). We included “1” as an addend (one problem within each subset), as well as the possibility to repeat the same addend in a problem (e.g., 4 + 4; four problems within each subset) to increase the pool of possible problems.

**Table 1:** Examples of the problems presented in the computation task (commutative and noncommutative subsets) and (in Experiments 2 and 3) the judgment task.

computation task				judgment task	
commutative subset		noncommutative subset		2 + 7 + 9	◯
				9 + 5 + 4	◯
Exp. 1	Exp. 2 and 3	Exp. 1	Exp. 2 and 3	**2 + 6 + 5**	◯
				**6 + 5 + 2**	◯
2 + 3 =	3 + 5 + 4 =	3 + 2 =	5 + 3 + 4 =	8 + 7 + 5	◯
**6 + 5 =**	**4 + 9 + 8 =**	5 + 6 =	8 + 9 + 4 =	**3 + 5 + 6**	◯
**5 + 6 =**	**4 + 8 + 9 =**	9 + 2 =	6 + 7 + 8 =	**6 + 5 + 3**	◯
4 + 3 =	6 + 2 + 5 =	3 + 4 =	5 + 2 + 6 =	2 + 9 + 5	◯
**9 + 7 =**	**9 + 7 + 2 =**	7 + 9 =	2 + 7 + 9 =	**6 + 7 + 9**	◯
**7 + 9 =**	**2 + 7 + 9 =**	8 + 8 =	9 + 4 + 5 =	**9 + 6 + 7**	◯

Problems in bold indicate the commutative pairs of the respective task.

#### Procedure

The participants received all problems as paper-pencil tests in the classroom. An experimenter introduced all tasks to the whole group (of up to 25 children). Three to four additional experimenters observed small subgroups of up to five children within the larger group during the entire experiment. Children of different classes were distributed randomly to the different experimental conditions. For the approximation-first group, the experiment started with the approximation task followed by the computation task. The remaining children received the reversed order of tasks (computation-first group).

The approximation task began with a training sheet with one pair of 'candy problems'. The experimenter explained this problem exemplarily and solved the example together with the children. Once the children signalled that they had understood the instruction, they were asked to work through the 11 trials of the approximation task. According to a pilot study, time was limited to 1.5 min (enough time to solve all problems without calculating the results).

The computation task also started with a short instruction. Children were told to solve the problems as quickly and as accurately as possible. A warm-up phase with six addition problems followed (all were noncommutative). Children were given 2 min to calculate these problems (i.e., sufficient time to solve all six warm-up problems). After this short training, a second instruction followed. Children were informed that for the next two subsets, it would be impossible to solve all problems during the period of time given for each subset. The instruction also stressed that they should work through the problems page by page and from top to bottom. They also were told to work only with a pencil. The time limit for each subset was set to 3 min. After having finished the first subset, children paused for 1 min and then received the second subset without further instruction. By providing the same time limit for both arithmetic subsets and by keeping the difficulty of the problems comparable over both subsets, we assessed the use of the commutativity shortcut. A commutativity benefit should show in more problems per time being solved in the subset containing commutative problems compared to the subset not containing such shortcut options. On the contrary, in case of more problems solved in the second, noncommutative subset, a general practice effect rather than the exploitation of commutativity would be evident.

#### Design

The experimental condition (task order) and task format served as independent variables. Task format in the approximation task refers to Problem Type (commutative vs. noncommutative 'candy problems') and in the computation task to Subset (commutative vs. noncommutative subset). Our main dependent measure was the individual number of completed problems in each of the two computation subsets. The use of commutativity as a shortcut is mirrored in drawing back on the preceding calculation no matter if the result was right or not. Therefore, we decided to use the number of total solved problems as the dependent variable and not the number of correctly solved problems. For all statistical tests we used an alpha level of .05.

### 2.3 Results

We excluded the data of children who did not follow instructions, for example by missing to start working on the task or trying to calculate the approximation problems (two children in the approximation-first and two in the computation-first condition). Furthermore, children were excluded who solved less than three problems in one of the computation subsets (i.e., two standard deviations below the mean). This concerned two children in the approximation-first and two children in the computation-first group. Thus, 32 children remained in the approximation-first condition, and 28 in the computation-first condition. We will report the results of the computation task first and then summarize the results of the approximation task.

**Fig 3:**
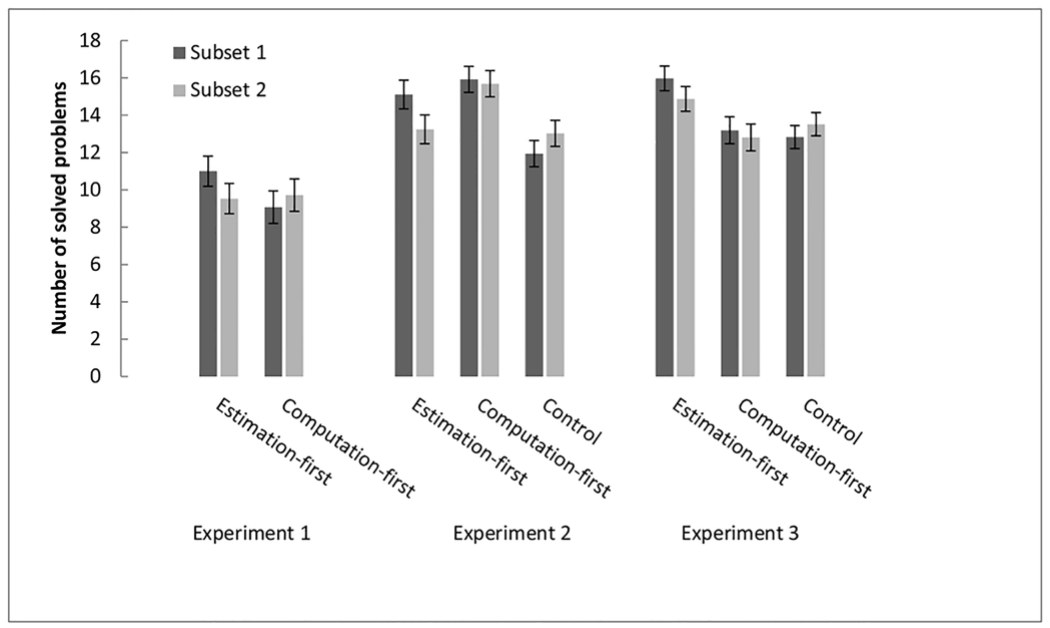
Numbers of solved exact arithmetic problems in Experiments 1, 2, and 3. Within experiments, numbers of solved problems are depicted as a function of subset and condition. In the approximation-first and the computation-first conditions, Subset 1 refers to commutative problems, and Subset 2 to noncommutative baseline problems. In the control condition, subsets 1 and 2 only contained noncommutative problems. Error bars reflect within-participants confidence intervals based on the MSe of the Condition X Subset Interaction [51].

To test for the effect of the approximation task on the computation task, we conducted a 2 x 2 mixed-design ANOVA with Condition as the between-subject and Subset as the within-subject factor and with number of problems solved within the given time as the dependent variable (see Fig 3). We chose to use number of solved problems as dependent variable rather than mean solution time per problem as the latter is more vulnerable to outliers. We are aware that this makes the comparison between the age groups difficult but it is sufficient for our main goal of within age-group comparison. We only found a significant interaction between Condition and Subset (*F*[1, 58] = 6.31, *MSe* = 5.28, *p* = .015, *η*_*p*_^*2*^ = .098, for all other effects *F* < 1). Planned contrasts indicated that the approximation-first group exhibited a substantial commutativity effect (*F*[1,58] = 6.54, *p* = .013, *d* = .33), whereas the computation-first group did not (*F* = 1.1, *p* = .30, see the left panel of Fig 3). The findings did not change when only including correctly solved problems in the analysis.

**Fig 4:**
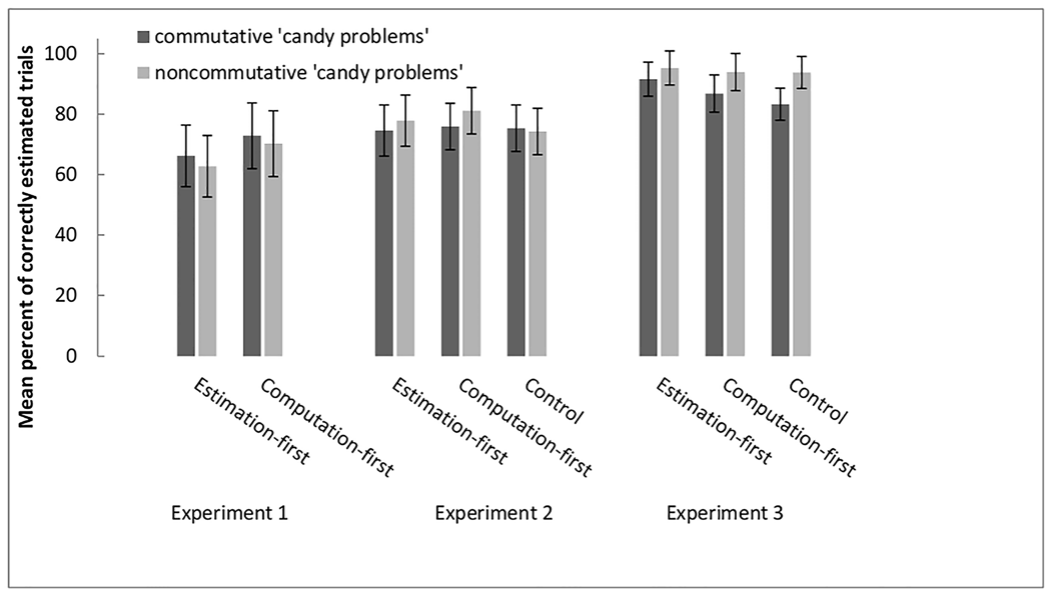
Percentage of correctly answered trials in the approximation task of Experiments 1, 2, and 3. Within experiments, commutative and noncommutative problems are depicted separately for the approximation-first, the computation-first and the control group. Error bars reflect within-participants confidence intervals based on the MSe of the Condition X Subset Interaction [51].

In order to investigate the effect of exact computation on the approximation task, we conducted a corresponding analysis for the approximation problems. The 2 (Condition) X 2 (Problem Type: commutative vs. noncommutative 'candy problems') mixed-design ANOVA with the proportion of correctly answered problems as the dependent variable did not yield any significant effect (all *F*s **<** 1, see also the left panel of Fig 4).

The results indicate that both conditions did not differ with regards to the approximation problems (see Table 2). Furthermore, commutative 'candy problems' were not easier to answer than the noncommutative problems, and the exact arithmetic tasks did not affect the performance in the approximation task [39].

**Table 2:** Performance of young first graders in the approximation task in Experiment 1.

N	Condition	*M* solved	% correct	% correct commutative	% correct non-commutative
32	approximation-first	6.81 *(2*.*46)*	65.15 *(30*.*84)*	66.18 *(40*.*56)*	62.76 *(34*.*91)*
28	computation-first	9.11 *(2*.*02)*	71.63 *(25*.*55)*	72.83 *(29*.*25)*	70.24 *(32*.*27)*

Mean number of solved approximation problems, rates of correct answers in general as well as separately for commutative and noncommutative 'candy' problems are depicted for both conditions (SD in parentheses). Participants had 1.5 min to solve 11 approximation problems.

### 2.4 Discussion

Experiment 1 revealed that an induction phase with commutative and noncommutative symbolic approximate calculation problems increased the first graders’ ability to spot and use the commutativity-based shortcut in exact addition problems. By contrast, we did not find the reverse effect from exact addition problems to approximation problems. As mentioned above, our approximation task was mainly constructed as an induction rather than as a measure of its own. Therefore, results concerning performance in the approximation task should be treated with caution (see also the discussion section).

Thus, approximation problems can not only enhance exact symbolic arithmetic performance [39], they can also trigger the subsequent use of an arithmetic principle by children in less familiar, abstract addition problems [39]. It is important to note that this was the case even though our participants had very little experience in formal addition and never had received any classroom instruction about the commutativity principle. Apparently activating precursory commutativity knowledge (i.e., knowledge about the order-irrelevance principle acquired before formal instruction in school) can help children to apply this knowledge to exact arithmetic problems. This raises the question of whether approximate calculation might also activate existing precursory conceptual knowledge, or if the influence is limited to the more general path of promoting flexibility in problem solving (procedural knowledge).

## Experiment 2

The second experiment aimed at testing the question of whether our approximation task either only influences procedural knowledge or, alternatively, can also trigger conceptual knowledge of the commutativity principle. This time we tested slightly older first graders who had attended school for approximately nine months. In contrast to the group of first graders in Experiment 1, these children had already received classroom instruction about the commutativity principle. Thus, participants of Experiment 2 should not only be trained in solving addition problems, but also should have (at least) some formal conceptual knowledge about commutativity (see for example [42]). In order to assess conceptual knowledge, we used the above mentioned *judgment task* ([48]; Table 1) as an additional task. It resembled the computation task as it contained 30 addition problems altogether; 18 of which formed 9 commutative pairs. In contrast to the computation task, children were instructed to only mark—without calculating the result of a problem—those problems that would require no calculation to get the result (i.e., the commutative problems). Again, children received no information about the existence of commutative problems. Consequently, only those students who possess conceptual knowledge—in the above mentioned sense, students who know why and under which conditions the procedure can be applied—should understand this instruction and should be able to identify the commutative problems.

Experiment 2 comprised three different tasks: (a) the approximation task, (b) the computation task, and (c) the judgment task. We expected to replicate the finding that activating precursory commutativity knowledge enhances the use of commutativity knowledge during exact calculation (i.e., in the computation task). If our induction with the approximation task also activates conceptual knowledge, we should observe an impact of administration of the approximation task on the number of correctly marked commutative problems in the judgment task. This was an open question, since Sherman and Bisanz [39] had not found any effect of their nonsymbolic task on conceptual knowledge.

As in Experiment 1, we realized the *approximation-first group* in which children started with the approximation task, and the *computation-first group* in which children began with the computation task. We also introduced an additional *control group*. In this group, children started with the two computation subsets. However, these two subsets did not contain any commutative problems. Participants were then given the approximation task and (like the approximation-first group) finished with the judgment task. The control condition had two functions: first, it helped us to assess the magnitude of the general practice effect when receiving two consecutive sets of arithmetic problems without the commutativity shortcut option. The computation-first group might have made use of the commutativity shortcut, but the benefit might have been occluded by a general practice effect. The control condition should allow us to differentiate the general speedup from problem subset 1 to problem subset 2 from the effect specific to the usage of commutativity knowledge. The second function was to measure the direct influence of the approximation problems on conceptual knowledge. In the approximation-first group, the effect of approximation problems on conceptual knowledge might be moderated by additionally encountering commutativity while calculating the problems of the computation task. Thus, there might be a direct and/or an indirect effect. However, as the control group received only noncommutative computation problems and, furthermore, worked through the approximation problems immediately before the judgment task, it exclusively measured the impact of the approximation task on the judgment task performance (conceptual knowledge).

### 3.1 Method

#### Participants

In Experiment 2, 131 first graders participated (55 girls, mean age of 7 years and 3 months, *SD* = 4.9 months). We recruited children from three elementary schools situated in middle socio-economic status suburbs of Cologne. Forty children (15 girls) were assigned to the approximation-first group, 45 children (17 girls) in the computation-first group, and 46 children (23 girls) were tested in the control group. All children had permission to join our study.

### 3.2 Procedure and Materials

The study was composed of three parts in which the three different tasks (approximation, computation, and judgment task) were administered. All tasks were conducted in one session (overall less than 45 min).

#### Materials

The *approximation task* was administered as reported for Experiment 1 with the only difference that this time it contained 14 trials (problem pairs), seven of which were commutative.

The *computation task* consisted of two subsets with 30 problems each. In contrast to Experiment 1, the problems here were *three*-addends addition problems with addends between 2 and 9 (maximum result was 24; 0 and 1 were excluded as addends). Three-addend problems were used to test whether our approximation task is suited to activate commutative knowledge to entirely unfamiliar commutative problems. To this end, we accept that three-addend problems imply associativity as well as commutativity (see also [19, 48]). Problems were spread over five pages, with six problems on each page. In the commutative subset, each page contained two problems that were commutative to their precursors (see Table 1). The noncommutative subset was constructed in the same way with the only exception that no commutative problems occurred.

The *judgment task* [48] also consisted of 30 three-addends-addition problems distributed over three pages. Among the 10 problems per page, three problems were commutative to their respective precursor problem (same addends in different order). The first page of the judgment task was for training. Participants were instructed to first compute the solutions of the problems on this page. Afterwards, they were instructed to mark those problems which they believed needed no calculation to obtain the correct solution (e.g. [44], for similar instructions]. For the remaining two pages, children were told not to calculate but instead only mark those problems they believed needed no calculation. Therefore, problems on these pages were presented without equal signs. Instead, a circle to the right of the problems could be marked (see Table 1). Bermejo and Rodriguez [52] found that among 6–7 year-olds, less than 10% needed to actually calculate results to discriminate commutative from noncommutative expressions. So, we are confident that children—provided they possess conceptual knowledge about commutativity—are able to recognize commutative problems in that format.

#### Procedure

The procedure was similar to Experiment 1. This time each participant received four booklets, containing the approximation task, the two subsets of the computation task, and the judgment task, respectively.

The *approximation-first group* started with the approximation task. Children were instructed to solve as many 'candy problems' as possible. The time limit was 2 min. A short break (2 min) followed. Afterwards the computation task started. All children began with the commutative subset and then received the noncommutative subset without any further instruction. The time limit was set to 4 min per subset (1 min more than in Experiment 1, as children here received three-addends problems). As in Experiment 1, the judgment task was presented after a one minute break. Participants received 2 min to solve as many problems as possible of the 10 addition problems on the first (training) page. They were reminded in the instruction that they should work through the page from top to bottom again. Almost all children completed the 10 problems before reaching the time limit. The experimenter then explained that some of the problems just calculated could alternatively have been solved without calculation. Children were asked to look for such problems and to mark them when they felt they could get the answer without actually calculating the result. Again, participants had 2 min for that. After this training page, participants received the remaining two pages of this task. They were told not to solve the problems, but only to mark those that need no calculation. The time limit here was set to 3 min.

The *computation-first group* received the same three tasks in a different order. They started with the computation task, followed by the judgment task. Lastly, they received the approximation task.

The *control group* also started with the computation task. However, children here received solely noncommutative problems in *both* subsets. That is, they did not encounter any commutative problems in the computation task at all. Afterwards, children were given the approximation task and lastly worked through the judgment task.

#### Design

As in Experiment 1, the independent variables were experimental condition (task order) and task format: approximation task (Problem Type: commutative vs. noncommutative 'candy problems'), computation task (Subset: subset 1 vs. subset 2), and the judgment task. As the control group did not receive commutative problems in the first or second subset, we only refer to 'commutative' and 'noncommutative subset' when reporting on the approximation-first or computation-first group. Again, our main dependent variable was the number of completed problems in the two subsets of the computation task. As dependent measures in the judgment task, we measured hits (correctly identified commutative problems), false alarms (incorrectly marked problems), as well as sensitivity index d’ and response bias c from Signal Detection Theory (SDT). According to SDT, the sensitivity index d’ refers to the ability to discriminate between signal-present trials (the commutative problems) and signal-absent trials (the noncommutative problems). That is, the index d’ reflects the relation between hits (correctly marked commutative problems) and false alarms (FA, incorrectly marked noncommutative problems; d’ = z(Hits)–z(FAs)). The response bias c measures participants’ general tendency to mark problems as commutative (c = -0.5 x (z(Hits) + z (FAs))). In our study, a response bias is liberal if a child marks many commutative and noncommutative problems as problems that need no calculation. The response bias is conservative if a child only marks very few commutative and noncommutative problems as needing no calculation [53].

### 3.3 Results

Again, children who did not follow instructions or who solved remarkably few of the arithmetic problems (less than two SDs below the group means) were excluded from further analyses. In addition, we also excluded children who marked each problem in the judgment task (11 children in the approximation-first group, 10 children in the computation-first group, and 11 children in the control group). This led to 29 remaining children in the approximation-first group, 35 children in the computation-first group, and 35 children in the control group. Analyses with the unadjusted sample did not differ substantially from results reported in 3.3 and can be found in the S1 Appendix. As in Experiment 1, we first present the results of the computation and the approximation task, followed by the results of the judgment task. Finally, we describe the relationship between procedural and conceptual knowledge.

#### Computation task

The middle panel of Fig 3 depicts the number of problems solved in each of the two subsets for each of the three conditions. A 3 (Condition) x 2 (Subset) mixed-design ANOVA with number of solved problems as dependent variable yielded a significant main effect of Condition (*F*[2, 96] = 5.00, *MSe* = 38.48, *p* = .009, *η*_*p*_^*2*^ = .094), as well as a significant interaction between Condition and Subset (*F*[2, 96] = 7.97, *MSe* = 4.32, *p* = .0006, *η*_*p*_^*2*^ = .142). There was no main effect of Subset (*F*[1, 96] = 1.27, *MSe* = 4.32, *p* = .27). The main effect of Condition was due to children in the computation-first group solving more problems than the participants in the control condition (revealed by Scheffé Test, *p* = .009, *d* = 0.77). This difference was unexpected. It might be due to a sampling error, even though children from different classes were randomly assigned to the experimental conditions.

More importantly, planned interaction contrasts (Condition X Subset) revealed that the approximation-first group and the control condition differed significantly in the number of problems solved in the first vs. second subset (*F*[1, 96] = 15.93, *MSe* = 4.32, *p* < .001; *d* = 0.82). The comparison between the computation-first and the control condition just failed the level of significance (*F*[1, 96] = 3.49, *MSe* = 4.32, *p* = .065, *d* = 0.381). In addition, also the interaction contrast between the approximation-first and the computation-first condition was significant (*F*[1, 96] = 4.89; *p* = .029; *d* = 0.453). Thus, the expected Condition X Subset interaction again indicates that the approximation-first group benefited much more from the commutative problems than the computation-first condition. An additional analysis with only correctly solved problems as the dependent variable did not change the results.

#### Approximation task

The 3 (Condition) X 2 (Problem Type: commutative vs. noncommutative 'candy problems') mixed-design ANOVA with the proportion of correct responses in the approximation task as the dependent variable revealed no significant effects (each *F* < 1; see middle panel of Fig 4). This finding indicates that all conditions performed equally well on the approximation problems. Practice on calculation problems apparently did not affect the performance in the approximation task (see Table 3). Again it has to be kept in mind that the approximation task was constructed as an induction, not as an instrument to measure estimation competencies. As it comprised only few commutative vs. noncommutative candy problems, we have to be cautious regarding its reliability.

**Table 3:** Performance of older first graders in Experiment 2 and third graders in Experiment 3 in the approximation task.

	Condition	*M* solved	% correct	% correct commutative	% correct non-commutative
Experiment 2					
n					
29	approximation-first	11.86 *(2*.*66)*	76.16 *(22*.*09)*	74.58 *(34*.*34)*	77.87 *(19*.*61)*
35	computation-first	12.14 *(2*.*60)*	78.95 *(20*.*81)*	75.91 *(31*.*80)*	81.12 *(23*.*55)*
35	control group	11.23 *(2*.*71)*	75.49 *(27*.*52)*	75.37 *(34*.*71)*	74.25 *(25*.*96)*
Experiment 3					
n					
31	approximation-first	13.39 *(1*.*48)*	93.37 *(9*.*04)*	91.55 *(14*.*26)*	95.31 (8.66)
35	computation-first	13.81 *(0*.*57)*	90.38 *(14*.*70)*	86.81 *(23*.*88)*	93.96 *(9*.*19)*
26	control group	13.43 *(1*.*4)*	88.56 (*16*.*33)*	83.31 *(28*.*40)*	93.81 *(10*.*62)*

Mean number of solved approximation problems, rates of correct answers in general as well as separately for commutative and noncommutative 'candy' problems are depicted for both conditions (SD in parentheses). Participants had 2 minutes to solve 14 approximation problems.

#### Judgment Task

For the conceptual knowledge task, we first computed the hit rate (proportion of correctly identified commutative problems) and false alarms rate (proportion of incorrectly marked noncommutative problems) for each child individually. In addition, we computed the sensitivity index d’ and the response criterion c. Table 4 depicts these results.

**Table 4:** Performance of older first graders in the judgment task in Experiment 2.

Condition	Hits	False alarms	Sensitivity d’	Response criterion c
approximation-first	.78 *(*.*24)*	.49 *(*.*32)*	1.76 *(1*.*76)*	-.56 *(1*.*21)*
computation-first	.69 *(*.*35)*	.42 *(*.*29)*	1.47 *(1*.*68)*	-.24 *(1*.*47)*
control group	.58 *(*.*35)*	.28 *(*.*31)*	1.63 *(2*.*37)*	.32 *(1*.*23)*

Rate of hits and false alarms as well as the sensitivity index and the response criterion for each condition are depicted (SD in parentheses).

As can be seen from Table 4, the mean d’ values did not differ very much between conditions. The corresponding one-way ANOVA with Condition as independent and d’ values as dependent variable revealed no significant effect (*F* < 1). However, a closer look on the hit and false alarm rates in Table 4 also showed that the *general* frequency of marking problems in the judgment task varied considerably between the groups. Therefore, we also compared the conditions’ mean response criteria. The response criterion c reflects participants’ response bias with negative c scores indicating liberal, positive scores a conservative response bias. The one-way ANOVA showed a substantial effect of Condition on participants’ response criterion (*F*[2, 96] = 3.72, *MSe* = 1.73, *p* = .028, *η*_*p*_^*2*^ = .072). A Scheffé Test revealed that the response criterion differed significantly between the approximation-first and the control group (*p* = .033, *d* = -0.72). Children in the approximation-first condition responded more liberally than children in the control condition. Thus, overall, the findings of the judgment task suggest that our approximation task, albeit it affected procedural knowledge, did not influence the conceptual knowledge about commutativity [39].

#### Relation between procedural and conceptual knowledge

As indicators of procedural knowledge, we used the number of solved problems in the commutative and the noncommutative subset, as well as the difference scores between the two subsets. We included hit rate, false alarm rate, and the sensitivity scores (d’) for measures of conceptual knowledge. An integrated concept of commutativity should be indicated by a significant correlation between the difference score and the sensitivity score d’. The control condition was excluded from this analysis since these participants did not receive any commutative problems in the computation task. Thus, no measure of procedural knowledge exists for this condition. Table 5 presents the correlations for the two experimental groups separately, as well as collapsed across both conditions.

**Table 5:** Correlation coefficients between procedural and conceptual knowledge in Experiment 1 and Experiment 2.

		**computation task**			
	**judgment task**	Subset 1	Subset 2	Difference	N
approximation-first & computation-first	Hits	.09	.01	.12	64
	False Alarms	.05	-.08	.21	
	d’	.16	.14	.01	
approximation-first	Hits	.06	.03	.04	29
	False Alarms	-.08	-.20	.21	
	d’	.20	.24	-.09	
computation-first	Hits	.14	.07	.13	35
	False Alarms	.20	.11	.16	
	d’	.15	.11	.08	
		**computation task**			
	**judgment task**	Subset 1	Subset 2	Difference	
approximation-first & computation-first	Hits	.09	.01	.12	
	False Alarms	.05	-.08	.21	64
	d’	.16	.14	.01	
approximation-first	Hits	.06	.03	.04	
	False Alarms	-.08	-.20	.21	29
	d’	.20	.24	-.09	
computation-first	Hits	.14	.07	.13	
	False Alarms	.20	.11	.16	35
	d’	.15	.11	.08	

Correlation coefficients between procedural and conceptual knowledge for first graders, depicted separately for all participants and for the three conditions of Experiment 2. Subset 1 contains the commutative problems, Subset 2 the noncommutative problems.

As can be seen from Table 5, both experimental conditions showed only small and non-significant correlations between the difference score and d’. Even when collapsing both conditions in order to increase power, no substantial correlation was detected.

### 3.4 Discussion

Experiment 2 yielded two main results: first, we could replicate our findings of Experiment 1. In comparison to the control group, both the approximation-first and the computation-first conditions demonstrated at least some procedural knowledge of commutativity as measured in the computation task. However, the approximation-first condition profited significantly more from commutative problems than the computation-first condition. Thus, Experiment 2 again indicated that the approximation task facilitated the use of the commutativity shortcut when solving exact arithmetic problems.

Second, this benefit was restricted to procedural knowledge—as it was in the study of Sherman and Bisanz [39, 54]. Conceptual knowledge was not enhanced. If at all, presenting the approximation task first slightly liberalized first graders’ response criterion in the judgment task. One possible explanation seems to be that the experience of not having to calculate mathematical problems exactly gave the children the impression that this can apply to more or less any arithmetical problem. However, we did not find this effect in the control condition in which approximation was administered directly before the judgment task without the intermediary computation. So, there seems to be no or at least no direct causal link from isolated estimation to judging more liberally afterwards.

Finally, we did not find a substantial correlation between the application of the commutativity shortcut and conceptual knowledge in the approximation-first condition or in the computation-first group, as reflected in children’s sensitivity in the judgment task. On the one hand, this result suggests that our approximation task is not suitable to boost the integration of both knowledge types in first graders. On the other hand, this missing correlation additionally supports the findings of Canobi et al. [19, 44] or Haider et al. [48] who found first measureable integration of procedural and conceptual commutativity knowledge in second or third graders. Therefore, to maximize the chance of finding a possible beneficial influence of our approximation task on integration of conceptual and procedural knowledge in Experiment 3, we tested whether our approximation task might affect conceptual knowledge in third graders.

## Experiment 3

The main goal of Experiment 3 was to replicate our findings with third graders. In addition, we tackled the question of if our approximation task would enhance conceptual knowledge when participants possess more basic conceptual knowledge about commutativity, and, furthermore, if it is suited to boost the integration of procedural and conceptual knowledge about the principle. Haider et al. [48] found first signs of an integrated concept of commutativity among third graders. Furthermore, Baroody [55] reported that third graders are even able to generate estimation strategies for unpractised problems based on the law of commutativity for multiplication problems. Therefore, we surmised that if our approximation task affects conceptual knowledge about commutativity, we should be able to find a similar effect in this age-group.

### 4.1 Method

#### Participants

One hundred and six (58 girls) third graders with a mean age of 8 years and 6 months (*SD* = 10 months) were recruited from three primary schools located in different middle socio-economic status suburbs of Cologne. Thirty-six (20 girls) children participated in the approximation-first group, 32 (15 girls) children in the computation-first group, and 38 (23 girls) in the control group.

### 4.2 Procedure and Materials

With the exception of time limits, materials and procedure were identical to Experiment 2. That is, we assigned children to one of three groups: the *approximation-first*, *computation-first*, and *control condition*. The time limit in the approximation task was unchanged; the other time limits were adopted for the third graders according to time demands estimated based on Haider et al. [48]. That is, we granted 3 minutes to solve each of the two computation subsets and 2 minutes for the judgment task.

#### Design

Independent variables again were experimental condition (task order) and task format: approximation task (Problem Type: commutative vs. noncommutative 'candy problems'), computation task (Subset: Subset 1 vs. Subset 2), and judgment task. The individual proportion of correctly solved problems in the approximation task, the number of solved problems in both subsets of the computation task, and the proportion of hits and false alarms in the judgment task served as dependent variables. Again, we additionally computed d´ and c from signal detection theory.

### 4.3 Results

Employing the reported exclusion criteria, the data of five children in the approximation-first group, of six children in the computation-first group, and of three children in the control group were excluded from further analyses. This led to 31 remaining children in the approximation-first group, 26 in the computation-first group and 35 in the control group.

#### Computation Task

A 3 (Condition) X 2 (Subset) mixed-design ANOVA with the number of solved problems as dependent variable revealed a significant main effect of Condition (*F*[2, 89] = 3.53, *MSe* = 31.51, *p* = .034, *η*_*p*_^*2*^ = .073), as well as a significant Condition X Subset interaction (*F*[2, 89] = 3.89, *MSe* = 3.42, *p* = .024, *η*_*p*_^*2*^ = .08). Fig 3 shows that the effect of Condition was due to the approximation-first group solving more problems than the other two conditions. As in Experiment 2, we assume that this is a sampling effect.

Planned interaction contrasts (Condition X Subset) only revealed a significant difference between the approximation-first and the control group (*F*[1, 89] = 7.64, *p* = .007, *d* = 0.59; *F*s < 1.5 for the other two contrasts). As in the previous experiments, the results did not change when we restricted our analysis to only correctly solved problems.

#### Approximation Task

The corresponding 3 (Condition) X 2 (Problem Type: commutative vs. noncommutative 'candy problems') mixed-design ANOVA for the approximation task only revealed a main effect of Problem Type (*F*[1, 89] = 9.26, *MSe* = .02, *p* = .003, *η*_*p*_^*2*^ = .094). This finding was due to more correctly solved noncommutative problems. Thus, third graders did not show a positive influence of the computation problems on the approximation task either—at least not with an approximation task set up as an induction rather than as a sensitive measure.

#### Judgment Task

Table 6 shows the hit and false alarms rate, as well as the sensitivity scores d’ and the response criterion c. A one-way ANOVA on d’ scores yielded no significant effect of Condition (*F* < 1). Thus, the three conditions did not differ regarding their sensitivity in the judgment task.

**Table 6:** Performance of third graders in the judgment task of Experiment 3.

Condition	Hits	False alarms	Sensitivity d’	Response criterion c
approximation-first	.75 *(*.*21)*	.38 *(*.*32)*	1.85 *(2*.*32)*	-.24 *(*.*98)*
computation-first	.72 *(*.*31)*	.28 *(*.*32)*	2.47 *(2*.*81)*	.11 *(1*.*01)*
control group	.71 *(*.*27)*	.30 *(*.*30)*	2.30 *(2*.*06)*	-.002 *(1*.*08)*

Again, the data of hit and false alarm rates suggests that children in the approximation-first group tended to respond more liberally than children in the other two conditions. However, the one-way ANOVA with the response criterion c as the dependent variable showed no significant differences between the conditions (*F* < 1).

Altogether, also third graders benefited from the approximation task when asked to solve arithmetic commutative and noncommutative problems. This finding is less clear than in the former two experiments, as the interaction contrast between the approximation-first and the computation-first conditions was not significant. However, only the approximation-first group differed significantly from the control group and only in this group children solved significantly more problems in the first (the commutative subset), compared to the second subset (the noncommutative subset; *F*[1, 89] = 5.46, *p* = .023, *d* = 0.24; *F* < 1 for the computation-first group). As in the former experiments, this positive effect of the approximation task was restricted to procedural knowledge only. Even though third graders possessed more conceptual knowledge than first graders (measured in terms of the sensitivity index d’ in the judgment task), we did not succeed in fostering their conceptual commutativity knowledge by means of symbolic approximation problems.

#### Relation between procedural and conceptual knowledge

As we did in Experiment 2, we also analyzed if procedural and conceptual commutativity knowledge is related. A positive correlation between these types of knowledge would indicate the formation of an increasingly abstract concept. Table 7 depicts the correlation coefficients for the approximation-first and the computation-first group, as well as collapsed across both conditions. As can be seen from Table 7, the correlations between the difference scores (procedural knowledge) and the sensitivity scores d’ (conceptual knowledge) are small and non-significant among third graders as well.

**Table 7:** Correlation coefficients between procedural and conceptual knowledge for third graders.

		computation task			
	judgment task	Subset 1	Subset 2	Difference	N
approximation-first & computation-first	Hits	.09	.04	.09	57
	False Alarms	.08	.14	-.12	
	d’	-.03	-.08	.10	
approximation-first	Hits	.17	.11	.04	31
	False Alarms	-.07	.06	-.22	
	d’	.11	.02	.14	
computation-first	Hits	.01	-.08	.13	26
	False Alarms	.16	.19	-.02	
	d’	-.11	-.18	.09	

Correlation coefficients between procedural and conceptual knowledge for third graders, depicted separately for all participants and for the three conditions of Experiment 3. Subset 1 contains the commutative problems, Subset 2 the noncommutative problems.

### 4.4 Discussion

The pattern of results in Experiment 3 is quite similar to that found for the younger children in Experiments 1 and 2: first, children who started with the approximation task were more likely to spot and apply the commutative shortcut in the computation problems than children who received the computation task at first. By contrast, the reverse effect, a potential impact of solving arithmetic problems on approximation, was not observed.

Second, third graders’ conceptual knowledge was not altered by the approximation induction. While the d’-scores of third graders were higher than those of first graders, reflecting a higher familiarity with the commutativity principle of addition, approximation problems had no impact on later judging whether or not arithmetic problems could be solved without computation. The liberalization effect found for the approximation-first condition in Experiment 2 was only descriptively replicated in Experiment 3. Consequently, as the effect does not seem to be very robust, we do not provide further speculations. However, it might be worthwhile to take up this issue in future research.

Again, we did not find any sign of a better integration of procedural and conceptual commutativity knowledge in the approximation-first condition. Thus, our data once again suggests that the approximation induction solely enhanced the application of the commutativity shortcut when solving arithmetic problems.

## General Discussion

In three experiments, we explored if symbolic approximate arithmetic can increase the subsequent spontaneous usage and understanding of commutativity in exact symbolic arithmetic problems as encountered in school. There is now growing consensus that children with little numerical experience are able to master nonsymbolic or symbolic approximate addition problems with large addends as long as no exact calculation is required (e.g. [24, 32, 56]). In addition, some recent findings suggest that elementary school children can benefit from approximate nonsymbolic problems in their subsequent performance on exact symbolic arithmetic problems [38]. However, we know of only a few studies that investigated potential effects of approximate arithmetic on the understanding of more abstract arithmetic principles as, for instance, inversion or commutativity [39]. Here, we focused on the question of whether activating precursory commutativity knowledge through approximate arithmetic problems will boost the exploitation of commutativity-based shortcuts in exact arithmetic problems [9].

Our study yielded three main results: first, the approximation task in fact increased the probability for children to apply the commutativity shortcut in exact arithmetic problems. Furthermore, we tentatively conclude that this influence seemed to be unidirectional since there was no comparable effect of solving arithmetic problems on the approximation task. Second, even though conceptual knowledge of commutativity improved from first to third graders, approximation had no positive effect on conceptual knowledge. Third, the positive effect of commutativity-related approximation on spotting and applying commutativity-based shortcut options in exact arithmetic problems was already observed in children who had not yet received any classroom instruction about the commutativity principle in school.

Our findings suggest that letting children explore the mathematical principle of commutativity in approximation problems activated some procedural precursory mathematical knowledge [26–27]. This activation was sufficient to trigger the usage of the shortcut during calculation. That is, when confronted with the approximation problems, children might have realized or might have been reminded that an important strategy in arithmetic is to attend to the addends and to compare them within and between problems [40]. If addends are identical, the results of the problems are also identical. Applying a shortcut strategy like that differs from understanding the abstract mathematical concept of commutativity in that it does not necessarily refer to the cardinality principle. It also does not necessitate metacognitive awareness of that one is no longer calculating when solving the problems either. The shortcut might simply be recognized as a helpful and labour-saving strategy when children are asked to calculate problems that follow the commutativity principle. This assumption might explain why we found reliable transfer from approximation to exact computation problems (procedural knowledge of commutativity), but not from approximation to judging arithmetic problems (conceptual knowledge of commutativity). Thus, we conclude that our approximation induction mainly triggered procedural knowledge and increased the flexibility of applying different strategies.

Our findings are in line with those of Matthews and Rittle-Johnson [57] who activated procedural knowledge via instruction and subsequently found a strategy transfer to unfamiliar problems, but no gains in conceptual knowledge. Also Sherman and Bisanz [39] or Fyfe, Rittle-Johnson, and DeCaro [58] did not find any transfer from procedural to conceptual knowledge. Therefore, it seems justified to conclude that letting children explore a principle on the level of quantities at first (that is, with reference to everyday objects and without demanding an exact answer, see [9]) is effective for boosting the usage of correct computational strategies and principle-based shortcuts that facilitate respectively supersede computation. However, the activated knowledge does not seem to be conceptual in the sense of an explicit representation of the abstract mathematical principle of commutativity (see also [39]). It might be that exploration of commutativity activates implicit knowledge representations rather than explicit conceptual knowledge about the abstract principle, or that the activation of the strategy after our induction is simply not sufficient for the children to become consciously aware of it [12, 59].

As an alternative interpretation of our results, one could argue that the impact of our approximation induction was simply due to an unspecific warm-up effect in the approximation-first conditions. The computation-first conditions in our experiments always started with the commutative subset of the computation task, whereas the approximation-first conditions received this subset after having solved the approximation problems. Thus, an unspecific warm-up effect seems plausible and would also explain the missing effect of our induction on conceptual knowledge as both groups would have been 'warmed-up' at this point. However, two arguments speak against this alternative explanation: first, an unspecific warm-up effect should have increased the overall number of problems solved in the approximation-first group compared to the computation-first condition. Obviously, this was not the case in our experiments: the conditions in Experiment 1 did not differ at all in the overall number of arithmetic problems solved. In Experiment 2, the computation-first group solved more problems, and only in Experiment 3, it was the approximation-first group. Second, in an additional experiment from our lab [60, 61], we compared the effects of different commutativity-based induction tasks on subsequent strategy-use in second graders. If such an induction phase serves as a general warm-up, one should expect no difference between these groups. However, the exploitation of the commutativity shortcut in the computation task did not occur in all of the conditions—although they all should have provided ample opportunity for warm-up and, more importantly, each of the inductions contained commutative problems. Thus, the alternative assumption of an unspecific warm-up effect seems rather unlikely to explain the current findings.

Why then is conceptual knowledge unaffected by the approximation induction? And why is there little integration of conceptual and procedural knowledge? So far, there seems to be no consensus on how to foster the development of abstract mathematical concepts. Our results suggest that experience with formal instructions in school does not seem to 'do the job'. Neither the first graders in our Experiment 2, who had explicitly been taught the commutativity principle some months before participating in our experiment, nor the third graders of Experiment 3, who had received such instruction two years before and afterwards spent much time on practicing basic arithmetic, showed any stable relationship between their strategy use and their ability to recognize the commutative problems in the judgment task, nor did they perform at ceiling level in these tasks. This is especially noteworthy given the findings of McNeil and Alibali [62]. They found that focusing on practice and correctly applied procedures during the initial learning of a mathematical principle led to less direct benefits in conceptual knowledge in third- and fourth-grade children than when conceptual guidance was involved. Interestingly, some weeks later the procedural conditions of the experiments had caught up in their conceptual understanding! So it is possible that a conceptual gain from the application of specific strategies needs much more time than what was given in our experiments (see also [63]).

Another account for our results could be that our judgment task was not reliable and/or the according instruction might have been misleading. However, Haider et al. [48] tested the reliability of the instrument and found satisfying split-half reliability coefficients between .78 and .83 for elementary school children. Also, the second argument—the instruction to the judgment task might have been misleading—does not seem to apply. First, when instructing the children, there was no indication that they did not understand the instruction. Second, there actually were some children in each sample who displayed perfect sensitivity in the judgment task and the number of these children increased from first to third graders. Of course, it is possible that some children drew on different ideas and concepts in trying to master the judgment task, but only relying on the principle in question—the additive law of commutativity—would result in the right answers and thus be measured as conceptual knowledge. Our material did not incorporate any other shortcut option that would result in a comparable benefit like exploiting commutativity. Third, Haider et al. [48] collected data of adult students. These participants showed near perfect knowledge in the judgment task. Given these arguments it seems justified to conclude that conceptual knowledge might emerge at a later point in development and could develop independent of procedural knowledge. The missing correlation in the current experiments was indeed due to the fact that children in the current study who were able to correctly mark all commutative problems did not show large benefits of commutativity in the computation task. Vice versa, children who showed large benefits of commutativity during calculation were not necessarily able to correctly mark the commutative problems in the judgment task. Thus, it seems that, at least in our study, the competencies assessed in the arithmetic and the judgment tasks are more or less independent.

Our findings additionally showed that first graders in Experiment 1, who never had been taught the commutativity principle in a formal context before, already benefited from the approximation task. This adds to the findings of Fyfe et al. [58] who studied the interplay of exploration and instruction in second and third graders. The authors found that the explicit instruction of a novel principle led to a higher usage of procedural knowledge when children could explore the task material without any feedback beforehand (as compared to when feedback was provided during exploration). Note that this was only the case for children who demonstrated some strategy knowledge before the exploration. So, it seems plausible that precursory procedural knowledge enabled our participants to benefit from the approximation task that was also administered without further guidance. This indicates that even an abstract arithmetic principle like commutativity, for which children possess informal precursory knowledge from everyday life, can be induced without any verbal explanation. Further support comes from the upper mentioned experiment from our lab (cf. [60]). The results showed that an explicit verbal explanation about commutativity alone did not elicit a larger procedural benefit than the approximation induction and, more importantly, this explicit instruction did not foster conceptual knowledge either.

Several findings already provided evidence for the existence of precursory knowledge in mathematics, and suggested that children understand basic arithmetic (i.e., addition or subtraction) as long as the tasks are carried out approximately [24–26, 28, 31–32, 56, 64–65]. In addition, there is evidence that these approximate competencies even predict later math performance in school [66–67]. A few other studies also indicated that activating precursory arithmetic concepts by inductions can facilitate children’s exact symbolic calculation performance (cf. [38]). For example, Obersteiner et al. [37] developed two analogue computer games that were aimed at strengthening either approximate or exact number processing skills in first graders. Children were trained either with one of the two versions or with a combination of both for a total of 5 hours. Besides a global training effect on children’s general mathematic abilities, the authors also found specific effects. The training on approximate number skills positively influenced the children’s performance in the approximation tasks (magnitude comparison, number comparison, and approximate calculation). In contrast, the training on exact number processing skills benefitted conceptual subitizing. Combining both trainings had no further advantage. Our current study extended these results by providing evidence that an approximation induction not only positively affects calculation and number processing, but also the spontaneous exploitation of a *specific abstract arithmetic principle* in an exact representational format even when no hint about its existence was provided beforehand.

Another important point regarding procedural knowledge is that, without the approximation induction, none of the conditions tested in the current studies displayed a benefit from commutative problems, at least not to a degree that led to significant differences between commutative and noncommutative problems. Even when comparing children who only received noncommutative problems (control group) with the computation-first condition, the benefit of commutative problems was not significant. Thus, this finding supports evidence that children up to third grade do not consistently spot and use the commutativity shortcut when receiving no hint about the existence of commutative problems [47, 68–70].

Altogether, the current results show that approximation problems can not only help to enhance general number processing or the execution of simple arithmetic [37–38], but also the use of a quite abstract and specific arithmetic principle. It strongly suggests that children already possess precursory knowledge about the principle of commutativity when entering school. They can rely on strategies derived from this knowledge, but seem to need external triggering to activate them, for instance with approximate calculation (e.g. [24]). Abstract conceptual knowledge in terms of an integration of conceptual and procedural knowledge about the commutativity principle seems to develop later and probably independent of such precursory knowledge (e.g., [35]). This might be one reason why it seems so difficult to enhance this conceptual knowledge.

### Limitations

There are limitations in our study that need to be discussed and improved. For example, future research should replicate our study with a more precise measure of approximate arithmetic. Our approximation task was developed primarily as an induction. With its ternary answer format, it provided a less precise measure than the computation and the judgment tasks. It would be helpful to test this hypothesis with an approximation measure equally sensitive than the exact ones to secure the finding of a unidirectional influence of approximation on exact arithmetic. In this case, a performance difference between commutative and noncommutative approximate problems can probably also be found, which would provide additional support for the assumption that approximation can trigger precursory commutativity knowledge.

This leads to the related question if it is the approximation alone or the inclusion of *commutative* approximation problems in our induction that triggered the procedural commutativity knowledge and increased flexibility in strategy use. In principle, noncommutative approximation problems could have been sufficient. We believe that the inclusion of commutative approximation problems is a crucial factor for the effect of the approximation task. This assumption is in line with the results of Fyfe et al. [58]. They found that exploring a specific principle without external feedback helped children with some prior strategy knowledge to better understand the subsequent instruction of this principle. It thus seems likely that the exploitation of this specific principle in our study at least in part goes back to its approximate representation in the induction. Nevertheless, this question should be followed up in future research by comparing the effects of our induction to the effects of an analogue approximation task that does not include the commutative problems.

Another aspect worth discussing is our assessment of procedural and conceptual commutativity knowledge. We tried to measure procedural and conceptual knowledge in an unobtrusive manner. Children received no direct hints concerning commutativity. We assessed procedural knowledge (‘knowing how’) with the computation task and, separately, conceptual knowledge (‘knowing why’) with the judgment task. It can be doubted that the computation task purely assessed procedural knowledge. If the measure also reflects conceptual knowledge though, performance in the computation and the judgment task should correlate [48]. We did not find such a correlation and hence conclude that mainly procedural knowledge was measured in our computation task that was not accompanied by conceptual knowledge. We believe that with our approach of assessing procedural and conceptual knowledge separately, we can rule out one critical aspect of the combined measurement: that it triggers the strategy use (see the description of [44] in 1.2). On the other hand, however, relying on a classroom setting and tasks providing no direct hints might have underestimated children’s conceptual knowledge. Future studies might administer our measures individually to increase reliability.

### Implications

Overall, our results show that activating children´s early knowledge of commutativity by a symbolic approximation task positively influenced the strategy use in formal arithmetic. This was the case either before or *after* children had received an according instruction in school. Therefore, we assume that children do not automatically activate their precursory mathematical knowledge in order to support their understanding of formal mathematical principles taught in primary school. However, our findings indicate that teachers can help children by explicitly referring to their informal knowledge. Although there is certainly a need to look for more methods and ways to improve the conceptual understanding of commutativity, it seems promising to include nonsymbolic, as well as symbolic approximate tasks as economical and practicable means in mathematical instruction. Our findings show that also Arabic numerals (symbolic number representation) can be used in approximation tasks [24]. While it might be feasible to induce mathematical principles nonsymbolically during the first year of school, this might seem like a setback to older children. Yet, in our study, approximation could successfully induce the exploitation of commutativity even in third graders. Thus, our findings suggest that symbolic approximation tasks can help children to spot and apply more efficient strategies in elementary school.

Summed up, our results suggest that inducing a principle in an approximate representation can help to link informal understanding in terms of Resnick’s [9] early levels of quantitative understanding to the understanding and exploitation of the numerical version of the principle. This might be an important premise for paving the way to an understanding of the principle in its truly abstract conception on the long run, enabling the learners not only to spontaneously use it, but also to integrate it in a broader context of more advanced mathematics.

## Supporting Information

S1 Appendix Experiment 2 unadjusted sample. (DOCX)

S1 Dataset Minimal Dataset of Experiments 1–3. (XLSX)
