# Supplementary material for: Fostering Formal Commutativity Knowledge with Approximate Arithmetic
Source: PLoS One. 2015 Nov 11;10(11):e0142551. doi: 10.1371/journal.pone.0142551 (PMC4652602; doi:10.1371/journal.pone.0142551)
Supplement: S1 Appendix — (DOCX) [file pone.0142551.s001.docx]

# Appendix

Statistical analyses of Experiment 2 conducted with the unadjusted sample

## Computation Task

A 2 (Condition) X 2 (Subset: commutative vs. noncommutative) ANOVA with number of solved problems within the given time as dependent variable revealed only a significant interaction between Condition and Subset (*F*[2, 128] = 9.8, *MSe* = 6.487, *p* = .0001, *η_p_^2^* = .133). The main effect for Condition (*F*[2, 128] = 1.61, *MSe* = 65.960, *p* = .204) and of Subset (*F* < 1) did not reach level of significance. Planned contrasts indicated that the approximation-first group exhibited a substantial commutativity effect (*F* = 9.99, *p* = .002, *d* = .27), whereas the computation-first group did not differ in number of solved problems between the subsets (*F* < 1) and the control group showed a substantial practice effect (*F*[1,128] = 8.93, *p* = .003, *d* = - .29), that is, more solved problems in the second than the first subset.

## Approximation Task

A 3 (Condition) X 2 (Problem Type: commutative vs. noncommutative 'candy problems') ANOVA with proportion of correct responses in the approximation task as dependent variable showed only a marginally significant effect of Problem Type (*F*[1, 127] = 3.82, *MSe* = 548.09, *p* = .053, *η_p_^2^*= .029). This effect was due to all conditions solving more of the noncommutative than commutative problems correctly. Both the main effect of Condition as well as the interaction did not reach level of significance (both *F*s < 1). Thus, findings in the unadjusted sample also suggest that all conditions performed equally well in the approximation problems and thus, practice on calculation problems did not affect performance in approximation (but remember that the approximation task was constructed as an induction rather than a measure).

## Judgment Task

The mean d´ values did not differ very much between conditions and mirrored interrelations as found in the adjusted sample; although on a lower level (see Table 8). This is not surprising given the fact that many of the excluded participants were taken out from analyses because they marked every single problem in the judgment task. Consequently, the corresponding one-way ANOVA with Condition as independent and d’-scores as dependent variable revealed no significant effect (*F* < 1). Analogous to our analyses in the adjusted sample, we additionally compared the conditions’ mean response criteria. The one-way ANOVA showed no effect of Condition on participants’ response criterion (*F*[2, 128] = 1.09, *MSe* = 2.517, *p* = .338), indicating that with inclusion of the participants who were observed to mark each problem in the judgment task, no specific response bias within the conditions was visible.

Table 8. Performance of older first graders in the judgment task in Experiment 2.

|  | d’ | | | |  | c | |
| --- | --- | --- | --- | --- | --- | --- | --- |
| Condition | Adjusted sample | n | Unadjusted sample | n |  | Adjusted sample | Unadjusted sample |
| approximation-first | 1.76 *(1.76)* | 29 | 1.56 *(1.71)* | 40 |  | -.56 *(1.21)* | -0.72 *(1.44)* |
| computation-first | 1.47 *(1.68)* | 35 | 1.29 *(1.75)* | 45 |  | -.24 *(1.47)* | -0.34 *(1.68)* |
| control group | 1.63 *(2.37)* | 35 | 1.35 *(2.22)* | 46 |  | .32 *(1.23)* | -0.24 *(1.61)* |

Mean sensitivity indices d’ and response criteria c for the different conditions of Experiment 2, in comparison for the adjusted as well as the unadjusted sample (the latter comprising the 32 excluded participants) are depicted (SD in parentheses).

Overall, the findings of the judgment task suggest that our approximation task, albeit it affected procedural knowledge, did not influence the conceptual knowledge about commutativity.
